# Andrographolide inhibits breast cancer through suppressing COX-2 expression and angiogenesis via inactivation of p300 signaling and VEGF pathway

**DOI:** 10.1186/s13046-018-0926-9

**Published:** 2018-10-12

**Authors:** Yulin Peng, Yan Wang, Ning Tang, Dongdong Sun, Yulong Lan, Zhenlong Yu, Xinyu Zhao, Lei Feng, Baojing Zhang, Lingling Jin, Fabiao Yu, Xiaochi Ma, Chuanzhu Lv

**Affiliations:** 1 Institute of Integrative Medicine, College of Pharmacy, College of Basic Medical Science, Dalian Medical University, Dalian, 116044 China; 2 Emergency Department, The Second Affiliated Hospital of Hainan Medical University, Haikou, 571199 China; 3 Department of Integrative Medicine, Liaoning University of Traditional Chinese Medicine Xinglin College, Shenyang, 110167 China

**Keywords:** Andrographolide, COX-2/NF-κB, Angiogenesis, p300, Breast cancer

## Abstract

**Background:**

Andrographolide (Andro), a diterpenoid lactone, has been used for treatment of various cancers with less adverse effects. However, the underlying mechanisms regarding its anti-tumor mechanism still remain unclear.

**Methods:**

Cell viability and proliferation were measured by CCK8 and CFSE dilution assay. The localization of p50/p65 or cytochrome c was determined using confocal immunofluorescence. Streptavidin-agarose pulldown or ChIP assays were used to detect the binding of multiple transactivators to COX-2 promoter. The promoter activity was examined by a dual-Luciferase reporter assay. The functions of Andro on COX-2-mediated angiogenesis were also investigated using human HUVEC cells through tube formation and spheroids sprouting assay. The in vivo anti-tumor efficacy of Andro was analyzed in xenografts nude mice.

**Results:**

The results indicated that Andro could significantly inhibit the proliferation of human breast cancers, and suppress COX-2 expression at both protein and mRNA levels. Furthermore, Andro could dose-dependently inhibit COX-2-mediated angiogenesis in human endothelial cells. We have also found that Andro significantly promoted the activation of cytochrome c and activated caspase-dependent apoptotic signaling pathway. Our further explorations demonstrated that Andro inhibited the binding of the transactivators CREB2, C-Fos and NF-κB and blocked the recruitment of coactivator p300 to COX-2 promoter. Moreover, Andro could effectively inhibit the activity of p300 histone acetyltransferase (HAT), thereby attenuating the p300-mediated acetylation of NF-κB. Besides, Andro could also dramatically inhibit the migration, invasion and tubulogenesis of HUVECs in vitro*.* In addition, Andro also exhibited effective anti-tumor efficacy as well as angiogenesis inhibition in vivo*.*

**Conclusion:**

In current study, we explore the potential effects of Andro in suppressing breast cancer growth and tumor angiogenesis, as well as the precise mechanisms. This work demonstrated the potential anti-cancer effects of Andro, indicating that Andro could inhibit COX-2 expression through attenuating p300 HAT activity and suppress angiogenesis via VEGF pathway, and thereby could be developed as an antitumor agent for the treatment of breast cancer.

## Background

Breast cancer is the most frequently diagnosed cancer in women, occupying nearly 15% in China [1] and 30% in U.S [2]. Because of its great invasiveness and malignancy, over 508,000 women might die of breast cancer a year in the world [3]. The primary treatment strategy for breast cancer is surgery and the following adjuvant chemotherapy. However, surgery could cause great damage on patients physically and psychologically [4], especially for young women, and the recurrence often occur in spite of adjuvant chemotherapy [5]. Current clinical management of breast cancer patients is also a great challenge due to the difficulty of designing individualized treatments, and drug resistance and dose-limiting toxicities could also hamper the therapeutic effect and prognosis of patients. Thus, it is very urgent to develop some novel therapeutic strategies and more effective agents to improve patients’ quality of life with less side effects.

Recently, the potential relationship between cyclooxygenase (COX-2) and cancer has become the hotspot in the anti-cancer research, and it has been demonstrated that COX-2 could play an important role in tumor promotion, especially breast cancer [6–8]. High expression of COX-2 can promote the proliferation, angiogenesis, invasiveness, metastasis, and also the inhibition of apoptosis and immuno- surveillance [9–11]. COX-2 expression could be transcriptionally regulated by the recruitment of trans-activators and co-activators to the corresponding sites of its promoters [12, 13]. Therefore, inhibition of COX-2 expression might be an effective way for the treatment of human cancer.

Histone acetyltransferase p300, known as p300 HAT or p300, is an enzyme that is encoded by the *EP300* gene in humans [14], and could be a transcriptional coactivator. P300 could be of great effect in regulating cell growth and division, and preventing the cancer growth. And p300 is usually expressed in relatively higher levels in cancer cells, compared with normal cells. P300 HAT activity regulates transcription of genes by binding to transcription factors [15], such as NF-κB, thereby enhancing transactivator binding [16]. Besides, p300 HAT has been demonstrated to be very essential for COX-2 promoter activation, which could significantly enhance the binding of transactivators [16, 17], and its downregulation could abrogate the stimulatory effect of various pro-inflammatory mediators on COX-2 expression. Thus, to find more effective agents especially natural compound, suppressing p300 HAT activity is regarded as an useful approach to promote the curation of COX-2-mediated diseases.

In recent years, increasing amounts of studies have focused on the natural products that exist in fruit and vegetables, due to their beneficial roles for human health. Andrographolide (Andro), a natural diterpenoid lactone which is isolated and identified from *Andrographis paniculata* that was used as traditional herbal medicine in many Asian countries for thousands of years. Andro has been reported to be of various biological activities, such as anti-inflammatory properties [18, 19] and anti-cancer properties [20, 21]. Previous studies have shown that Andro inhibited NF-κB activation and DNA binding activity [22, 23]. These studies strongly supported that Andro could be used as an effective agent for treatment of chronic inflammation-related disease including cancer.

Therefore, in present work, we aim to investigate the effect of Andro on COX-2 suppression and angiogenesis in human breast cancer cells in vivo and in vitro, and to explore whether Andro could target p300 signaling pathway to regulate COX-2 expression. Our findings fully indicated that Andro could serve as a potential candidate targeting p300 signaling pathway to suppress NF-κB activation for treatment of COX-2- mediated breast cancer.

## Methods

### Reagents and antibodies

Andrographolide (Andro) was isolated from *Andrographis paniculata* by our laboratory with its purity of 98.7%. In present study, Andro was dissolved in dimethyl sulfoxide (DMSO) as a 100 mM stock solution and stored at − 20 °C. Andro was diluted to obtain the desired concentration in cell culture medium, where the final concentration of DMSO was less than 0.1%. Control cultures received the carrier solvent (0.1% DMSO). The primary antibodies for COX-2, p-Cofilin, F-actin, cleaved-caspase 3/9, NF-κB p65 and p-p65, and all the secondary antibodies were obtained from Cell Signaling Technology (Cell Signaling Technology, Inc., USA). The primary antibodies for GAPDH, p300, and NF-κB p50 were obtained from Santa Cruz Biotechnology (Santa Cruz, CA, USA). The primary antibodies for Bax, Bcl-2, CD31, β-actin and cytochrome c were obtained from Proteintech Group (Proteintech Group, Inc., USA). Dulbecco’s Modified Eagle’s Medium (DMEM), RPMI 1640 and fetal bovine serum (FBS), trypsin were obtained from HyClone Laboratories (HyClone Laboratories Inc.). All other chemicals were purchased from Sigma Chemical Co. (St. Louis, MO) unless otherwise specified.

### Cell lines and cell culture

Human breast cancer cell lines MDA-MB-231, MCF-7, T47D, MDA-MB-361, and BT549 were obtained from the American Type Culture Collection (ATCC Manassas, VA, USA). These cells were cultured in Dulbecco’s Modified Eagle medium (DMEM) or 1640 medium supplemented with 10% bovine serum albumin (FBS), 100 μg/ml penicillin and 100 μg/ml streptomycin. Primary human umbilical vein endothelial cells (HUVECs) were isolated from human umbilical vein as described [24]. HUVECs were cultured in M199 containing 10% fetal bovine serum (FBS), 25 U/mL heparin, 5 ng/mL bFGF and 10 ng/mL EGF. The cells were cultured in a humidified atmosphere of 5% CO_2_ at 37 °C.

### Cell viability assay

Cell viability was determined using the CCK-8 assay. In brief, 5 × 10^3^ cells were seeded into 96-well culture plates allowed to adhere for overnight, and then the cells were changed to fresh medium containing various concentrations of Andro (5, 10, 25 and 50 μM) dissolved in DMSO (final concentration, less than 0.1%). After incubation for 48 h, CCK-8 was added, and the absorbance was measured at 4**5**0 nm by *EnSpire*® Multimode Plate Reade (Perkin Elmer, USA). Cell viability in vehicle control groups was considered 100%. Each assay was carried out at least in triplicate.

### Colony formation assay

To analyze the effects of Andro on colony formation, single cells (1 × 10^3^ per well) were seeded in six-well plate containing 2 ml growth medium with 10% FBS and cultured for 24 h. Then, removed the culture medium, and cells were treated with various concentrations of Andro. After 24 h, cells were washed with PBS and supplemented with fresh growth medium, and cells were routinely incubated for about 2 weeks until colonies were large enough to be visualized. Then colonies were stained with 0.1% crystal violet counted.

### Flow cytometry analysis

To determine the proportion of apoptotic cells and CFSE dilution, we performed flow cytometry analysis using a flow cytometer (BD FACS Accuri C6, CA, USA). For the apoptosis examination, the cells were washed with PBS, and stained using the Annexin V-FITC Apoptosis Detection Kit in the dark at room temperature for 15 min. The cell cycle distribution and the fraction of apoptotic cells were determined using a FACS analysis system. For CFSE dilution assay, CFSE (carboxyfluorescein succinimidyl ester) dilution was usually used to detect cell proliferation. Briefly, cells were stained with 5 μM of CFSE at 37 °C for 15 min followed by adding 5-fold medium with FBS to terminate the reaction. CFSE-stained cells were cultivated with Andro for 48 h, and then the cell division was analyzed by flow cytometry. Each experiment was performed in triplicate.

### Western blot analysis

Protein lysates were separated by electrophoresis in a 10% sodium dodecyl sulfate-polyacrylamide minigel (SDS-PAGE), electrophoretically transferred to a PVDF membrane, and immunoblotted with specific antibodies. The protein bands were detected by enhanced chemiluminescence. The protein concentrations were determined using a BCA protein assay kit (Beyotime Biotechnology, China). Similar experiments were carried out at least three times.

### Confocal immunofluorescence

In briefly, the cells grown on chamber slides with indicated Andro treatment were fixed with 4% paraformaldehyde and permeabilized with 0.2% TritonX-100. The samples were probed with specific antibodies against Cytochrome c (cyt c), p50 or p65 (Santa Cruz) and fluorescein isothiocyanate- and rhodamine-conjugated secondary antibodies. Subsequently, the stained samples were stained with DAPI to counterstain cell nuclei. The samples were examined under a Leica DM14000B confocal microscope.

### Streptavidin-agarose pulldown assay to detect DNA protein binding

Binding of NF-κB p65/p50 to COX-2 promoter probes was determined by a streptavidin-agarose pulldown assay. A 478-bp biotin-labeled double stranded probe corresponding to COX-2 promoter sequence (− 30 to − 508) was synthesized. Briefly, 400 ul mixture solutions containing nuclear extract proteins (400 μg) biotinylated DNA probe (4 μg), streptavidin-conjugated agarose beads (40 μl) and supplemented with PBSi (PBS buffer with 1 mM EDTA, 1 mM DTT and protease inhibitor cocktail complete) buffer at room temperature for 5 h on a rotating shaker. After washing with PBSi buffer, the beads were resuspended with the SDS-PAGE loading buffer and boiled at 100 °C. The supernatant was analyzed by Western blotting.

### Chromatin immunoprecipitation

The chromatin immunoprecipitation (ChIP) assay was performed as previously described. In brief, after treatment with Andro for 24 h, MDA-MB-231 cells were fixed with 1% paraformaldehyde for 10 min, and scraped in lysis buffer (1% SDS, 10 mM Tris–HCl, pH 8.0, with 1 mM phenylmethylsulfonyl fluoride, pepstatin A, and aprotinin). P50-specific antibody or control IgG antibodies were added into samples overnight at 4 °C. The protein A/G plus agarose beads were added into samples for DNA enrichment. DNA segment enrichment of COX-2 promoter (Forward primer: ACGTGACTTCCTCGACCCTC, and Reverse primer: AAGACTGAAAACCAAGC CCA) was examined by PCR.

### Dual-luciferase reporter assay

MDA-MB-231 cells in a 6-well plate, were co-transfected with pRL-TK plasmid (0.07 μg/well) and pGL3-NFκB plasmid (1.5 μg/well) or pGL3-basic plasmid (1.5 μg/well) (as negative control), which was encapsulated by Lipofectamine 2000 Reagent (3.75 μl/well) (Invitrogen, Grand Island, NY, USA). Meanwhile, cells were treated with Andro. After 48 h treatment, the dual-luciferase reporter assay system (Promega, Madison, WI) was used to measure the changes in luciferase activity. Firefly luciferase activity was normalized to Renilla luciferase activity. All values are shown as mean ± SD of triplicate samples.

### In vitro transfection assay

The MDA-MB-231 cells (1 × 10^6^) were plated in 60 mm dishes 1 day before transfection. The relevant plasmids encoding Flag-p300 (3 μg/dish) was transfected with the Lipofectamine 2000 transfection reagent (7.5 μl/dish) (Invitrogen), following the manufacturer’s instructions. After transfection, the cells were cultured for another 24 h. Subsequently, the transfected cells were trypsinized and seed in 60 mm dishes, followed by culturing with fresh medium containing indicated doses of Andro for 48 h, and then collected for indicated experiments.

### HAT activity assay

The HAT Activity was detected using the HAT Activity Colorimetric Assay Kit (Biovision,USA) following the manufacturer’s instructions. The nuclear extracts were prepared from the indicated cells treated with or without Andro.

### In vitro migration assay

Scratch assay (wound healing assay) was performed to detect cell migration ability. The HUVECs were grown to full confluence in six-well plates. Cell monolayers were wounded with a sterile 200 μl pipette tip and then washed with PBS after 6 h-starvation. The cells were changed to fresh medium (5% FBS) containing indicated doses of Andro, with or without 50 ng/mL VEGF. After 48 h, medium was replaced with PBS, the wound gap was observed, and cells were photographed using a Leica DM14000B microscope fitted with digital camera and the distance of the wound gap was measured.

### Transwell invasion assay

The motility of HUVECs was performed in 24-well transwell plates as previously described [25]. Briefly, the upper surface of polycarbonate filters with 8 μm pores was coated with 75 μL Matrigel (Matrigel: M199 = 1:3, without growth factors) and incubated for 0.5 h at 37 °C for gelling. Then, 5 × 10^4^ cells were seeded into the upper chambers and the bottom chamber were filled with 600 μL M199 with 10% FBS supplemented with VEGF (50 ng/ mL) or vehicle. Both top and bottom chamber contained the same concentrations of Andro. After 24 h incubation, non-invasive cells on the upper membrane surfaces were removed by wiping with cotton swabs. Invaded cells were fixed with methanol and stained with 0.1% Crystal Violet Staining Solution. The membrane was dried in the air. Images were taken using a Leica DM 14000B microscope. Cell invasion counted in five independent areas per membrane. The results were the means calculated from five replicates of each experiment.

### Tube formation assay

HUVECs [1 × 10^4^ in 50 μL M199 medium with 0.1% BSA containing Andro (0, 10, 20 μM), with or without 50 ng/mL VEGF] were seeded on Ibitreat angiogenesis slides (Ibidi, Martinsried, Germany) pre-coated with 10 μL Matrigel, and the formation of tubular like structure was recorded by a Leica DM14000B microscope at 6 h.

### Spheroids sprouting assay

Spheroids were generated by gravity as described [24]. Briefly, spheroids were embedded into a collagen gel, and then rapidly transferred into a 24-well plate and allowed to polymerize at 37 °C incubators for 30 min, M199 medium with or without VEGF and Andro was applied on top of the gel. After cultured for 24 h, spheroid sprouts were evaluated by measuring the cumulative length of all capillary like sprouts using a Leica DM14000B microscope. At least 5 randomly selected spheroids per experimental group and experiment were analyzed. And sprout length was measured with Image J software.

### Animal studies

Female athymic nude mice (BALB/c nu/nu, 4 weeks old, 18–20 g) were purchased from the SPF Laboratory Animal Center of Dalian Medical University (Dalian, China), and maintained in pathogen-free conditions at animal facility. MDA-MB-231 cells (1 × 10^7^ in 100 μL PBS) were injected subcutaneously near the axillary fossa of each nude mouse. Two weeks later, when the formed tumor reached about 30 mm^3^ after cell inoculation, the animals were divided randomly into three groups with 5 mice in each group, followed with Andro treatment (0, 5, 10 mg/kg) by intraperitoneal injection in 100 μl solution(PBS: Propylene Glycol: DMSO = 7:2:1, *v*/v)/per mouse, once every 2 days. Meanwhile, tumors were measured with a caliper once every 2 days, and the tumor volume was calculated using the formula V = 1/2 (width^2^ × length). Body weights were also recorded. After treatment for 15 days when the growth of tumor tissue in high dose (10 mg/kg) group has entered platform-phase, all experimental mice were sacrificed and the tumors from each mouse were excised and their weight was measured. To determine indicated proteins’ expression, the tumor tissues were fixed with 10% neutral formalin and embedded in paraffin. The sections (4 μm, from each tumor) were stained with COX-2 (1:100) antibody, and examined under a light microscope. To determine the microvessel density in tumor tissues, the sections (4 μm) were stained with CD31 (1:100) antibody, and examined under immunofluorescent microscope. The images were examined under a Leica DM 4000B fluorescence microscope equipped with a digital camera. The other parts of tumors were used to prepare tumor tissue lysates for Western blot analysis.

All animals were given free access to sterilized food and water and were habituated for a week before the experiments. All procedures were carried out in strict accordance with the recommendations established by Animal Care and Ethics Committee of Dalian Medical University as well as the guidelines by U.S. National Institutes of Health Guide for Care and Use of Laboratory Animals. The protocol was approved by the Animal Care and Ethics Committee of Dalian Medical University.

### Statistical analysis

All experiments were repeated at least three times. Data are represented as mean ± standard deviation (SD). One way analyses of variance or two-tailed Student’s t-test were used to compare the values of the test and control samples in vitro and in vivo. *P* < 0.05 was considered to be a statistically significant difference. SPSS 17.0 software was used for all statistical analysis.

## Results

### Andro suppressed the proliferation of human breast cancer cells

**Fig. 1:**
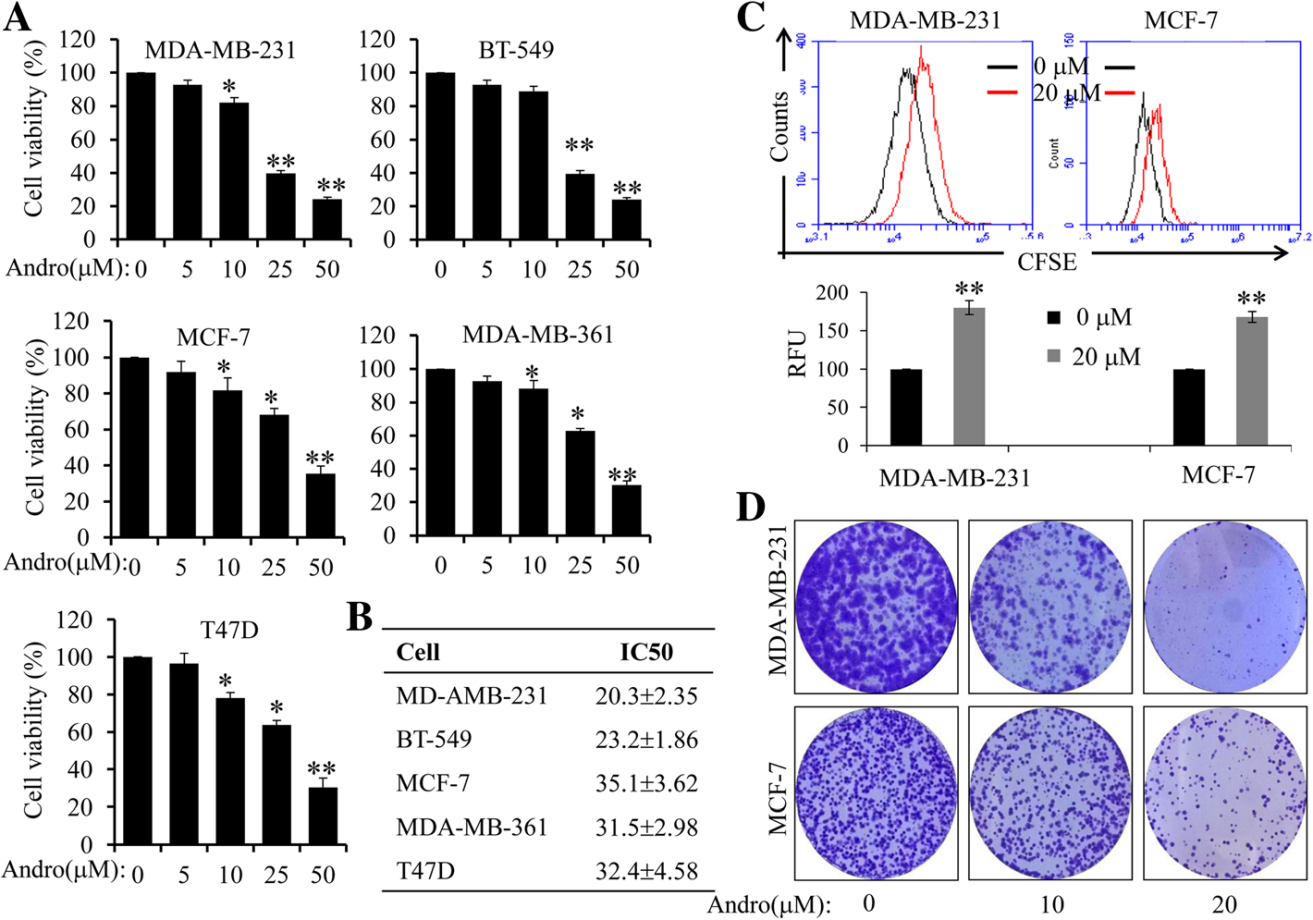
Effect of Andro on cell proliferation in human breast cancer cells. **a** Breast cancer cells (MDA-MB-231, MCF-7, T47D, MDA-MB-361, and BT549) were cultured with the indicated concentrations of Andro for 48 h, and then the cell viability was determined by a CCK-8 assay. **b** The IC_50_ values of Andro for cell viability inhibition in breast cancer cells were determined. **c** MDA-MB-231 and MCF-7 cells were stained with CFSE and cultured with Andro for 48 h. Cell proliferation was determined by a BD Accuri C6 Flow Cytometer. **d** MDA-MB-231 and MCF-7 cells were cultured with Andro. The induced colony formation was analyzed, and the colony formation numbers were calculated. The data are presented as the mean ± SD of at least three separate experiments. (*P < 0.05, **P < 0.01, Andro treatment vs vehicle control groups)

Firstly, the effects of Andro on cell proliferation were examined in several breast cancer cell lines by CCK-8 assay. These results indicated that Andro significantly inhibited the proliferation of various cancer cell lines, including MDA-MB-231**,** BT-549, MCF-7, MDA-MB-361 and T47D (Fig. 1a). The IC_50_ values of Andro for each breast cancer cell lines were also calculated. As shown in Fig. 1b, MDA-MB-231 cells were more sensitive to Andro treatment, compared with other cell lines. According to this, we selected MDA-MB-231 cells and two effective concentrations of Andro (10 μM, ~ 20% inhibition, 20 μM, ~ 50% inhibition), for further mechanistic studies. Furthermore, the effects of Andro on the proliferation were also determined by flow cytometry analysis of carboxyfluorescein succinimidyl ester (CFSE) dilution. Indeed, Andro markedly suppressed the proliferation of MDA-MB-231and MCF-7cells (Fig. 1c). We also evaluated the effect of Andro on the colony formation of MDA-MB-231 and MCF-7 cells. Results indicated that treatment with Andro also significantly suppressed the colony formation, compared with untreated groups (Fig. 1d). These results fully demonstrated that Andro exhibited significant anti-proliferative effects in human breast cancer cells.

### Andro promoted cell apoptosis by modulating cytochrome c and caspase signaling

**Fig. 2:**
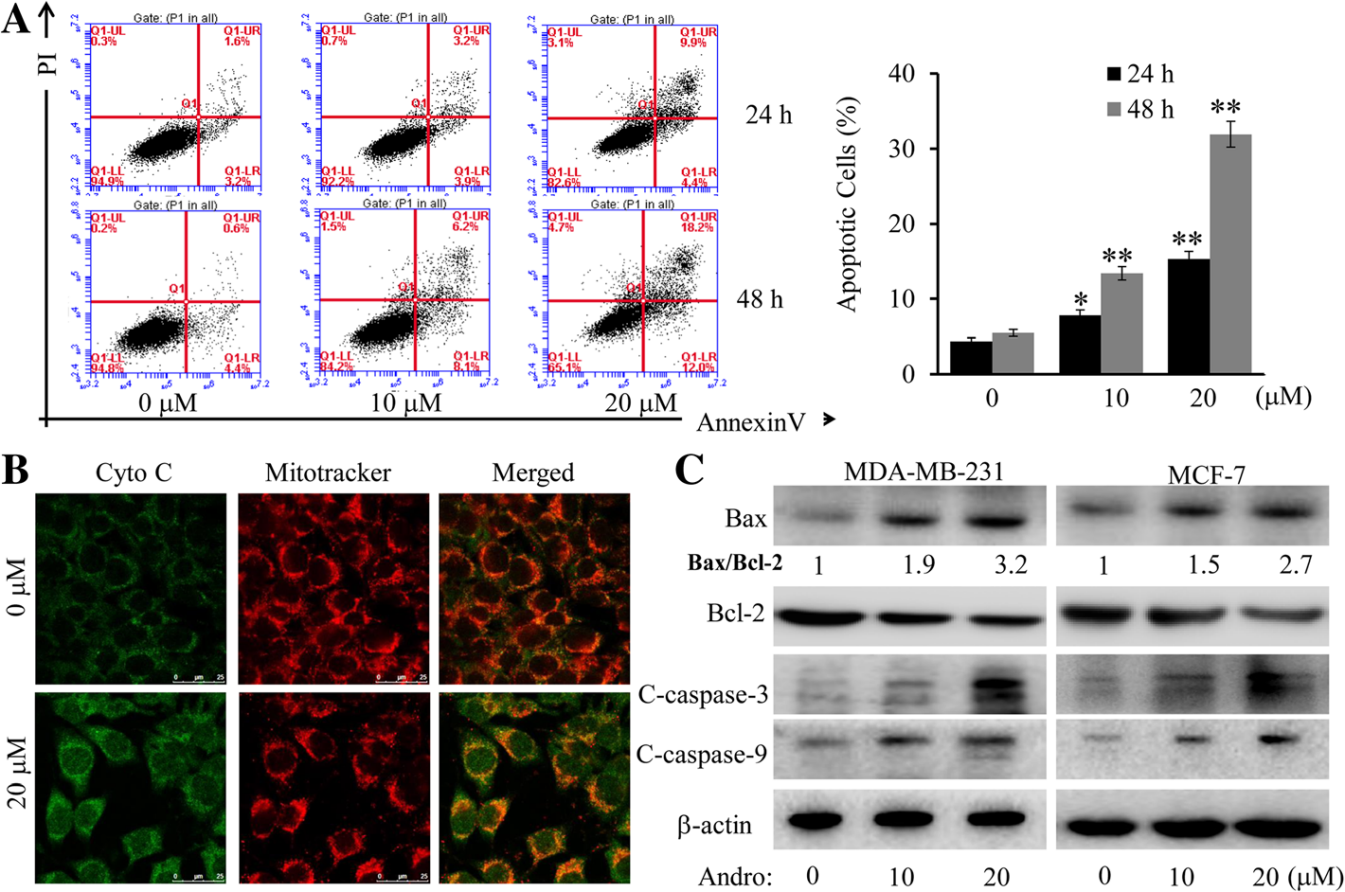
Effect of Andro on caspase-dependent apoptosis. MDA-MB-231 and MCF-7 cells were treated with Andro at the indicated doses for appropriate time. **a** The apoptosis was determined in MDA-MB-231 cells by a FACS analysis after treatment for 24 h or 48 h, and the percentage of apoptotic cells was calculated. **b-c** MDA-MB-231 cells were treated with Andro at the indicated doses for 24 h. The releasing process of cytochrome c from mitochondria to cytoplasm was observed by immunofluorescence imaging analysis (**b**). And the expression of the cleaved caspase-3/9 and Bcl-2/Bax proteins were analyzed by Western blot (**c**). Data were represented as the mean ± S.D. of at least three independent experiments. (*P < 0.05, **P < 0.01, Andro treatment vs vehicle control groups)

Apoptosis could be the therapeutic target for treatment of various cancers [26, 27]. By using FACS analysis, we seek to determine whether the cell growth inhibition induced by Andro could be associated with the activation of the apoptotic pathway. As shown in Fig. 2a, the results indicated that Andro dose- and time-dependently promoted the apoptosis from 4.9 to 15.1% at 24 h, and 5.4 to 31.95% at 48 h in MDA-MB-231 cells. Besides, it is well known that the translocation of cytochrome c (cyt c) from the mitochondrial intermembrane space to the cytosol could promote the cell apoptosis [28]. We thus exerted immunofluorescence imaging (IFI) analysis to confirm whether Andro could induce the release and translocation of cyt *c*. As shown in Fig. 2b, treatment of Andro markedly triggered the translocation of cyt c from the inter-mitochondrial space into the cytosol. Furthermore, to explore the detailed mechanisms underlying Andro-induced cell apoptosis, the levels of apoptosis-relative proteins (cleaved caspase-3/9, BAX and Bcl-2) in both MDA-MB-231and MCF-7 cells were also detected by Western blot after treatment for 24 h. As shown in Fig. 2c, Andro could markedly increase the protein levels of cleaved caspase-3/9, and the ratio of Bax/Bcl-2. These results demonstrated that Andro could induce cell apoptosis through triggering the release of cyt c from mitochondria, promoting the activation of multiple caspase cascades in the cytosol.

### Andro suppressed COX-2 expression through NF-κB signaling

**Fig. 3:**
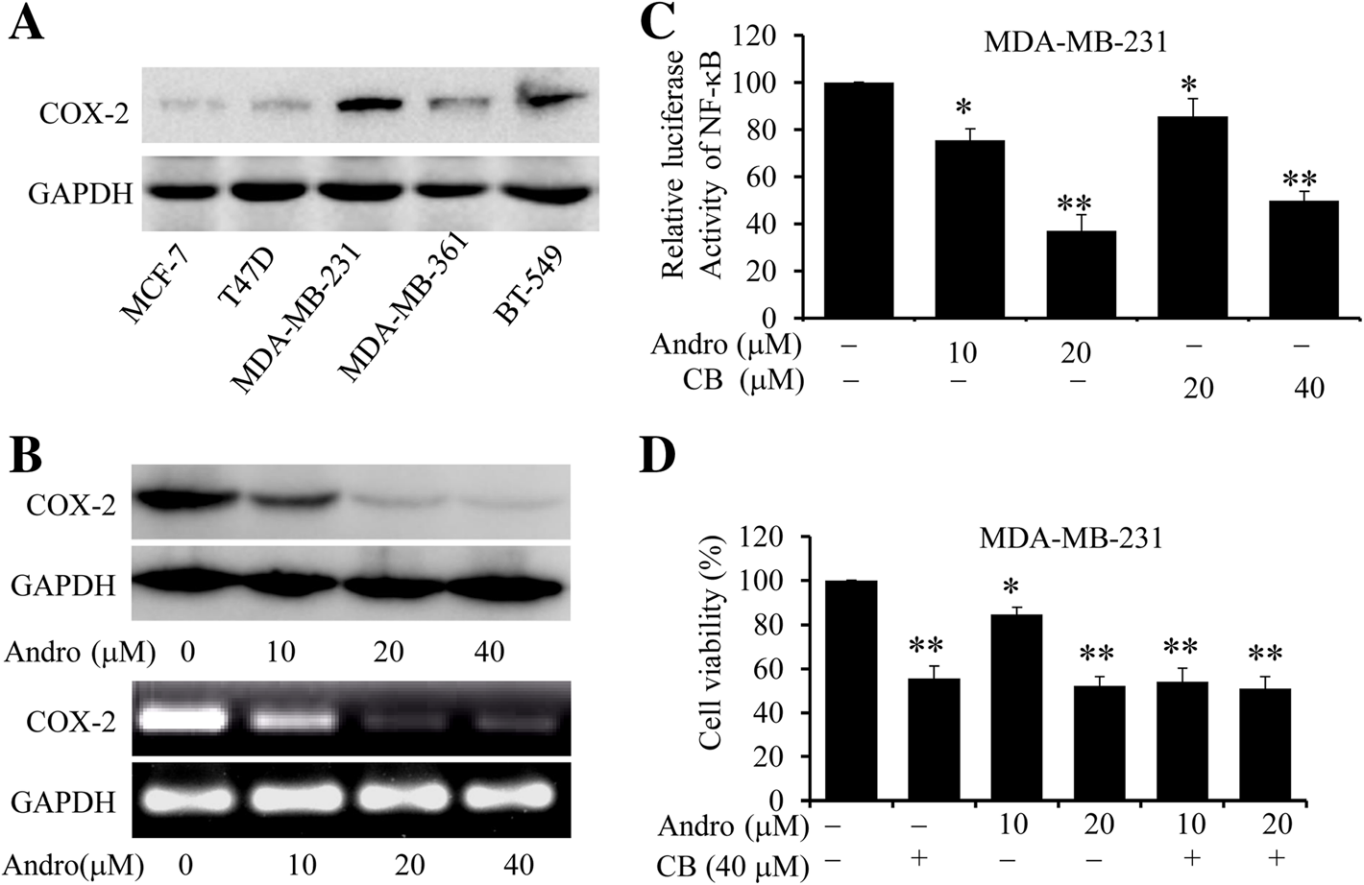
Effect of Andro on COX-2 expression in human breast cancer cells. The expression level of COX-2 protein was analyzed by Western blot in human breast cancer cells treated without **a** Andro treatment. **b** Expression levels of COX-2 protein and gene were analyzed by Western blotting (upper) and RT-PCR (bottom) in MDA-MB-231 cells treated with indicated doses of Andro for 48 h. **c** MDA-MB-231cells were cotransfected with pNFκB-luc with Renilla luciferase reporter (as internal control) for 24 h and then treated with Andro or CB for 48 h. Luciferase activity was determined using a dual-luciferase reporter assay system. **d** MDA-MB-231cells cells were treated with indicated doses of Andro for 48 h after pretreatment with the COX-2 selective inhibitor Celecoxib (40 μM) for 8 h, and the cell viability was determined by CCK-8 assay. Data were represented as the mean ± S.D. of at least three independent experiments. (*P < 0.05, **P < 0.01, Andro treatment vs vehicle control groups)

Overexpression of COX-2 has been demonstrated to promote cell proliferation and inhibit cell apoptosis in various cancer cells [6–8]. We next evaluated the regulating effects of Andro on COX-2 expression in human breast cancer cells. We first examined the expression of COX-2 in several breast tumor cells, including MDA-MB-231**,** BT-549, MCF-7, MDA-MB-361 and T47D. As shown in Fig. 3a, the expression of COX-2 was relatively higher in MDA-MB-231 cells than others. Furthermore, treatment of Andro significantly downregulate the expression of COX-2 at both protein and mRNA levels in a dose-dependent manner in MDA-MB-231 cells (Fig. 3b). Based on these results, we hypothesized that Andro could suppress COX-2 transcription by inhibiting the binding of some transcription factors to their promoter regions. We next evaluated the effect of Andro on NF-κB activity through a dual-Luciferase reporter assay. MDA-MB-231 cells were transfected with luciferase reporter plasmids containing NF-κB binding sites, following by the treatment with Andro or a COX-2-selective inhibitor celecoxib (CB, 20 and 40 μM). As shown in Fig. 3c, the activity of NF-κB signaling pathway was significantly decreased, after treatment with Andro or CB (positive group) in MDA-MB-231 cells. Besides, to further determine the regulating effect of Andro on COX-2 signaling, MDA-MB-231 cells were pretreated with a COX-2-selective inhibitor CB (40 μM) for 8 h, followed by treatment of Andro. Then the cell viability was determined by CCK-8 assay. As shown in Fig. 3d, treatment with CB or Andro alone exhibited different inhibitory effect on cell viability, whereas combined treatment of them did not significantly affect the inhibition of cell viability, compared with treatment alone. These results suggested that inhibitory effect of Andro on the proliferation of breast cells is mediated by regulating the activity of COX-2 signaling.

### Andro suppressed COX-2 promoter activation by inhibiting the binding of multiply trans-activators to COX-2 promoter

**Fig. 4:**
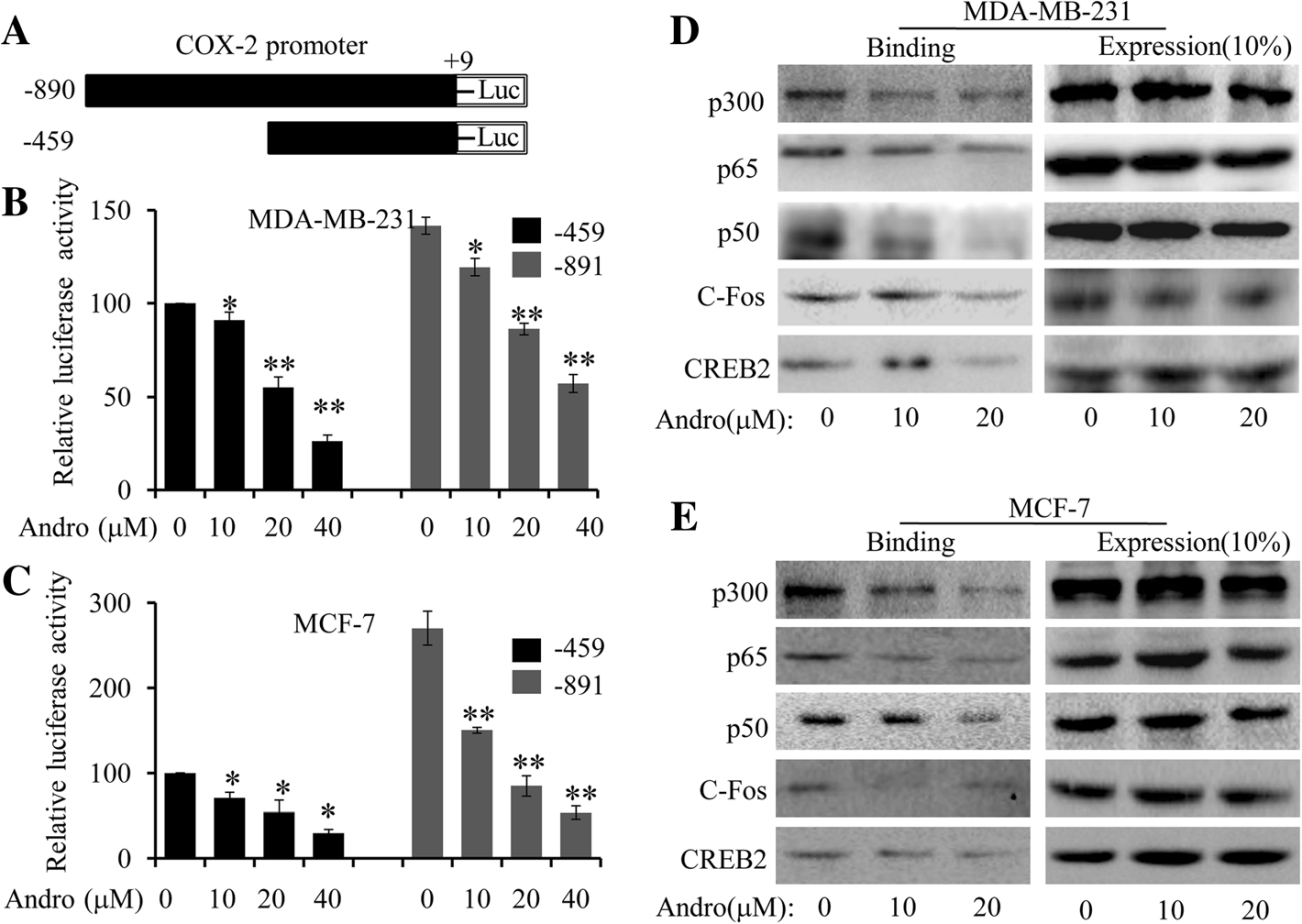
Effect of Andro on NF-κB signaling in human breast cancer cells. MDA-MB-231 and MCF-7 cells were cotransfected with a luciferase expression vector containing COX-2 promoter (− 890/+ 9 or − 400/+ 9) 5-flanking fragment (**a**) and Renilla luciferase reporter (as internal control) for 24 h and then treated with Andro for 48 h. Luciferase activity was determined using a dual-luciferase reporter assay system (**b** and **c**). **d** and **e** After 48 h treatment, the nuclear extract proteins were prepared and the binding of transactivators (CREB-2, C-Fos NF-κB p65 and p50) to COX-2 promoter probe was analyzed by a streptavidin-agarose pulldown assay. Data were represented as the mean ± S.D. of at least three independent experiments. (*P < 0.05, **P < 0.01, Andro treatment vs vehicle control groups)

The effects of quercetin on COX-2 transcription activation were determined by a dual-Luciferase reporter assay in two breast cancer cells. MDA-MB-231 and MCF7 cells were transfected with a luciferase expression vector containing COX-2 promoter (− 890/+ 9 or − 400/+ 9) 5-flanking fragment (Fig. 4a). The results showed that treatment of Andro significantly suppressed the activity of COX-2 promoter in MDA-MB-231 cells in a dose-dependent manner (Fig. 4b), which was parallel to variation trend of the changes of COX-2 protein and mRNA suppression (Fig. 3a and B). Similarly, promoter activity was also dose-dependently suppressed by Andro in MCF-7 cells (Fig. 4c).

By acting on the promoter region, COX-2 expression has been reported to be regulated by several transcription factors and transcriptional coactivator, including NF-κB [29], CREB-2, C-Fos and p300 [17]. To further determine whether Andro-mediated inhibition of COX-2 could be mediated by inhibiting the binding of functionally important transactivators to its promoter in human breast cancer cells, we next performed a streptavidin-agarose pulldown assay by using a 478-bp biotin-labeled double-stranded oligonucleotide probe containing COX-2 promoter sequence from − 30 to − 508. As shown in Fig. 4d and e, treatment of Andro dramatically inhibited the binding of these transactivators (CREB-2, C-Fos and NF-κB p50/p65) to COX-2 promoter region in a dose-dependent manner. However, Andro treatment could exert no effect on intracellular expression of all trans-activators (Fig. 4d and e). All these indicated that the inhibitory effect of Andro on breast cancer cells is partially mediated by inactivating the COX-2 signaling via suppressing the binding of muliple transactivators to the COX-2 promoter.

### Andro attenuated the activity of p300 HAT and p300-mediated acetylation of NF-κB

**Fig. 5:**
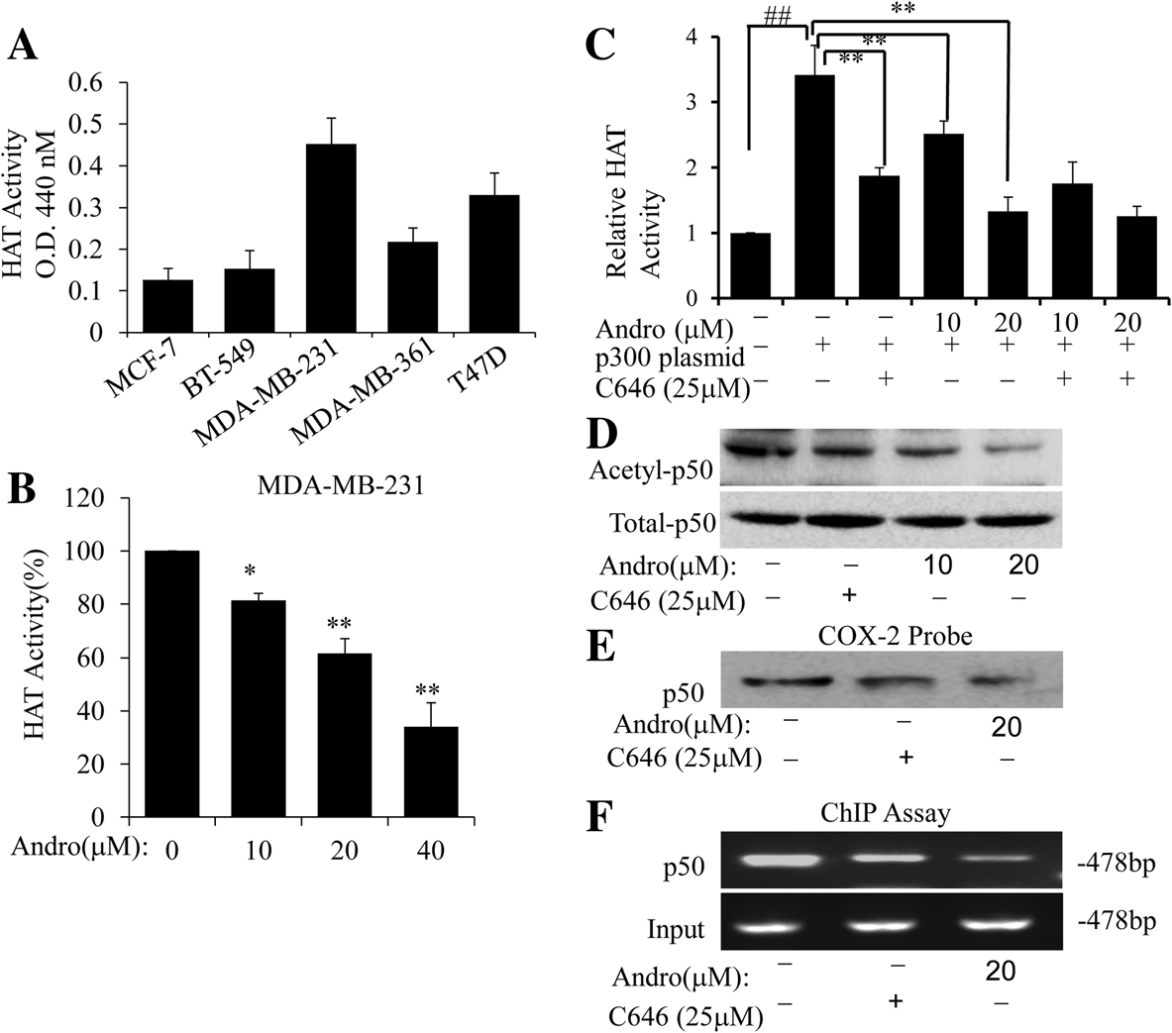
Effect of Andro on p300 HAT and acetylation of NF-κB in human breast cancer cells. **a** The HAT activities of human breast cancer cells were was measured. **b** MDA-MB-231 cells were cultured with indicated dose of Andro for 24 h. HAT activity was then determined. (*P < 0.05, **P < 0.01, Andro treatment vs vehicle control groups). **c** MDA-MB-231 cells were transfected with a p300 plasmid for 24 h, and then treated with Andro or C646 (p300 selective inhibitor) for 24 h. HAT activity was further determined. **d** The MDA-MB-231 cells were transfected with p300 plasmid for 24 h and then treated with Andro for 24 h. Nuclear extracts were prepared and p50 was immunoprecipitated with a p50 antibody. Acetylated p50 (Actyl-p50) **d** was analyzed by Western blots using an acetyl-lysine antibody. The binding of p50 to a biotinylated COX-2 promoter probe (**e**) and chromatin structure (**f**) were detected by streptavidin-agarose pulldown and ChIP assay, respectively. (##*p* < 0.01, p300 plasmid group vs control group, *P < 0.05, **P < 0.01, Andro treatment vs p300 plasmid groups)

It is known that oxidative stress can activate p300 HAT and then lead to the increased histone acetylation, regulating the gene expression like NF-κB [30–32]. The results indicated that Andro exerted inhibitory effect on the binding of NF-κB and p300 to COX-2 promoter, thus we hypothesized that p300 HAT might be a target of the transcriptional regulation of Andro in human breast cancer cells. To verify this hypothesis, we evaluated the effect of Andro on p300 HAT activity. Firstly, we tested the intracellular HAT activity of multiply breast cancer cells, including MDA-MB-231**,** BT-549, MCF-7, MDA-MB-361 and T47D. The results indicated that MDA-MB-231 cells could be of higher HAT activity than other cells (Fig. 5a). Next, MDA-MB-231 cells were cultured with indicated doses of Andro for 48 h, following by the detection of HAT activity. As shown in Fig. 5b, Andro significantly inhibited HAT activity in a dose-dependent manner in MDA-MB-231 cells. Furthermore, MDA-MB-231 cells were transfected with a p300 plasmid for 24 h, following by treatment of Andro or C646 (p300 selective inhibitor) for 24 h. HAT activity was then detected. Our results indicated that treatment of Andro or C646 could be of obvious inhibitory effect on the HAT activity, however, the combined treatment of Andro and C646 together could not markedly affect the HAT activity, compared with the treatment of Andro or C646 alone (Fig. 5c).

Previous studies have shown that p300 could acetylate the NF-κB p50, thereby increasing NF-κB-mediated transactivation [16, 33]. To further confirm the inhibitory effect of Andro on p300 HAT activity in human breast cancer cells, we thus evaluated the effect of Andro on NF-κB acetylation in p300-tranfected MDA-MB-231 cells. As shown in Fig. 5d, Andro significantly inhibited the p300 HAT-mediated acetylation of NF-κB p50, resulting in a marked reduction in acetyl-p50 protein levels. However, treatment of Andro had no significant effect on the expression of intracellular p50. Furthermore, by using streptavdin-agarose pulldown (Fig. 5e) and ChIP assay (Fig. 5f), we have also evaluated the effect of Andro on the binding activity of NF-κB p50 to COX-2 promoter in p300-tranfected MDA-MB-231 cells. Besides, compared with C646 (p300 selective inhibitor) treatment, Andro exerted more prominent inhibitory effect on the acetylation and binding activity of NF-κB p50 to COX-2 promoter (Fig. 5d, e and f). Above all, these results indicated that p300 HAT could be an important mediator for transcriptional regulation of Andro. And Andro could significantly inhibit p300 HAT activity and attenuate the acetylation and binding of trans-activators and suppress the expression of COX-2.

### Andro inhibited angiogenesis

**Fig. 6:**
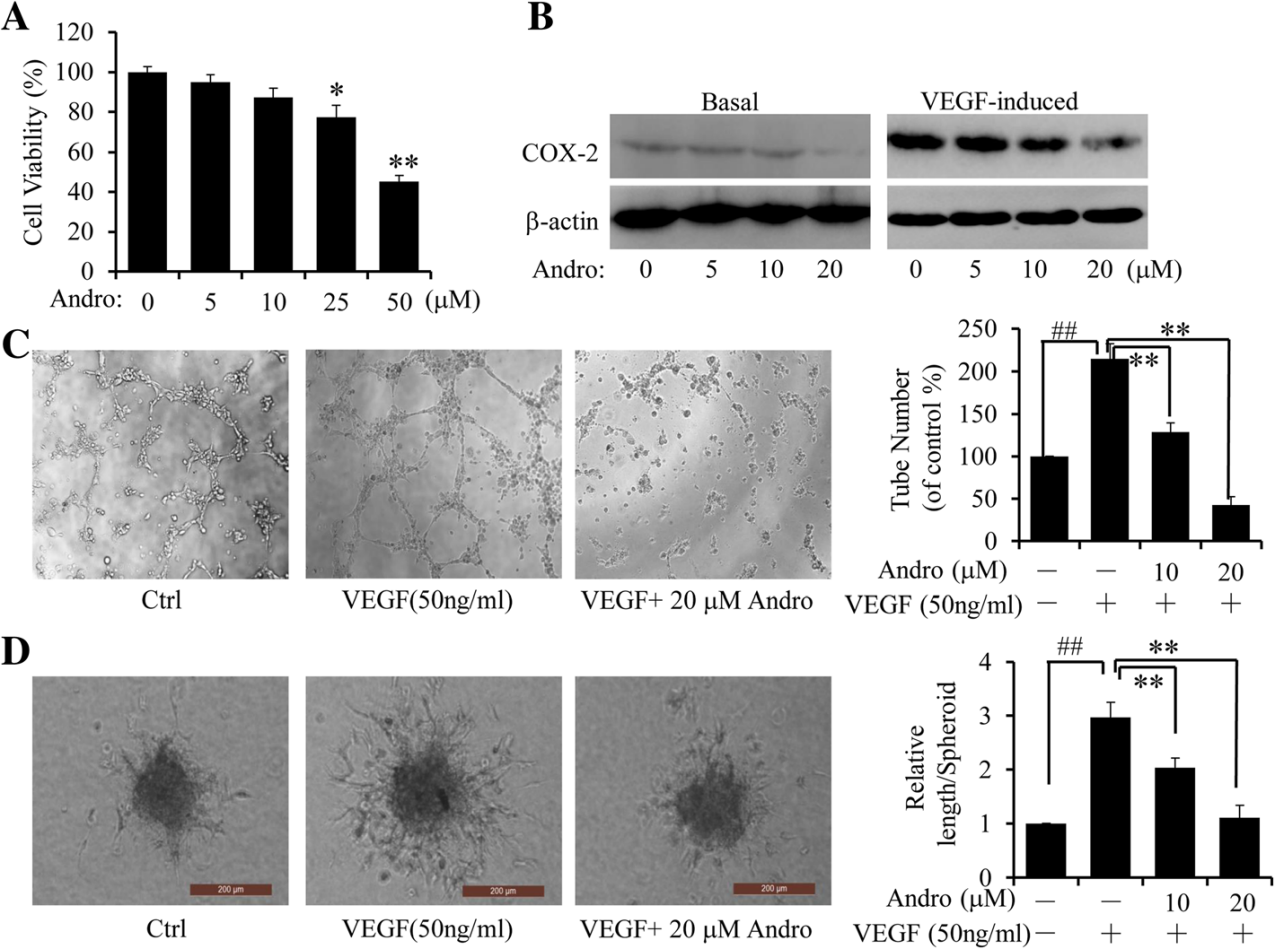
Effect of Andro on VEGF-induced angiogenesis. **a** HUVECs were exposed to Andro at the indicated doses, and viability was measured by CCK-8 assay. Data were represented as percentage of vehicle-treated control. **b** The expression level of COX-2 protein was analyzed by Western blot HUVECs treated with the indicated doses of Andro for 48 h, with or without VEGF induction. **c-d** Effects of Andro on tube formation on Matrigel **c** at 6 h (Original magnifcation, 50×), and sprouting from modifed human endothelial cell spheroids **d** at 24 h (Original magnifcation, 200×) . Experiments were performed with or without VEGF and indicated Andro doses. (##*p* < 0.01, VEGF-treated group vs. Solvent; *P < 0.05, **P < 0.01, Andro treatment vs vehicle control groups)

Since overexpression of COX-2 could be associated with increased angiogenesis and might be a maker of cell invasiveness in breast carcinoma [34], we thus hypothesized that Andro could suppress angiogenesis, which could promote the tumor growth and invasion [35, 36]. Inhibiting angiogenesis has been shown to be an effective strategy for suppressing tumour growth and metastasis [25, 37]. To verify this hypothesis, we selected human endothelial cell line HUVECs for further explorations. Firstly, we investigated the influence of Andro on the proliferation of human endothelial cells. As shown in Fig. 6a, treatment of Andro showed mild inhibitory effect on HUVECs proliferation and showed no obvious cytotoxicity at low concentrations. VEGF_165_ obviously induced COX-2 expression in HUVECs, while Andro dramatically suppressed the COX-2 expression induced by VEGF_165_ in HUVECs (Fig. 6b). Furthermore, by using an in vitro angiogenesis tube formation assay, we have also examined the effects of Andro on VEGF_165_-induced HUVECs tube formation. As shown in Fig. 6c, VEGF_165_ (50 ng/mL) significantly enhanced the endothelial capillary like structures, compared with control groups. However, this effect could be blocked by treatment of Andro in a dose-dependent manner. All these indicated that Andro could efficiently impair VEGF_165_-induced tube formation in HUVECs. Besides, it could also be determined that Andro significantly inhibited VEGF_165_-induced endothelial cell sprouting using a modified spheroid assay (Fig. 6d).

**Fig. 7:**
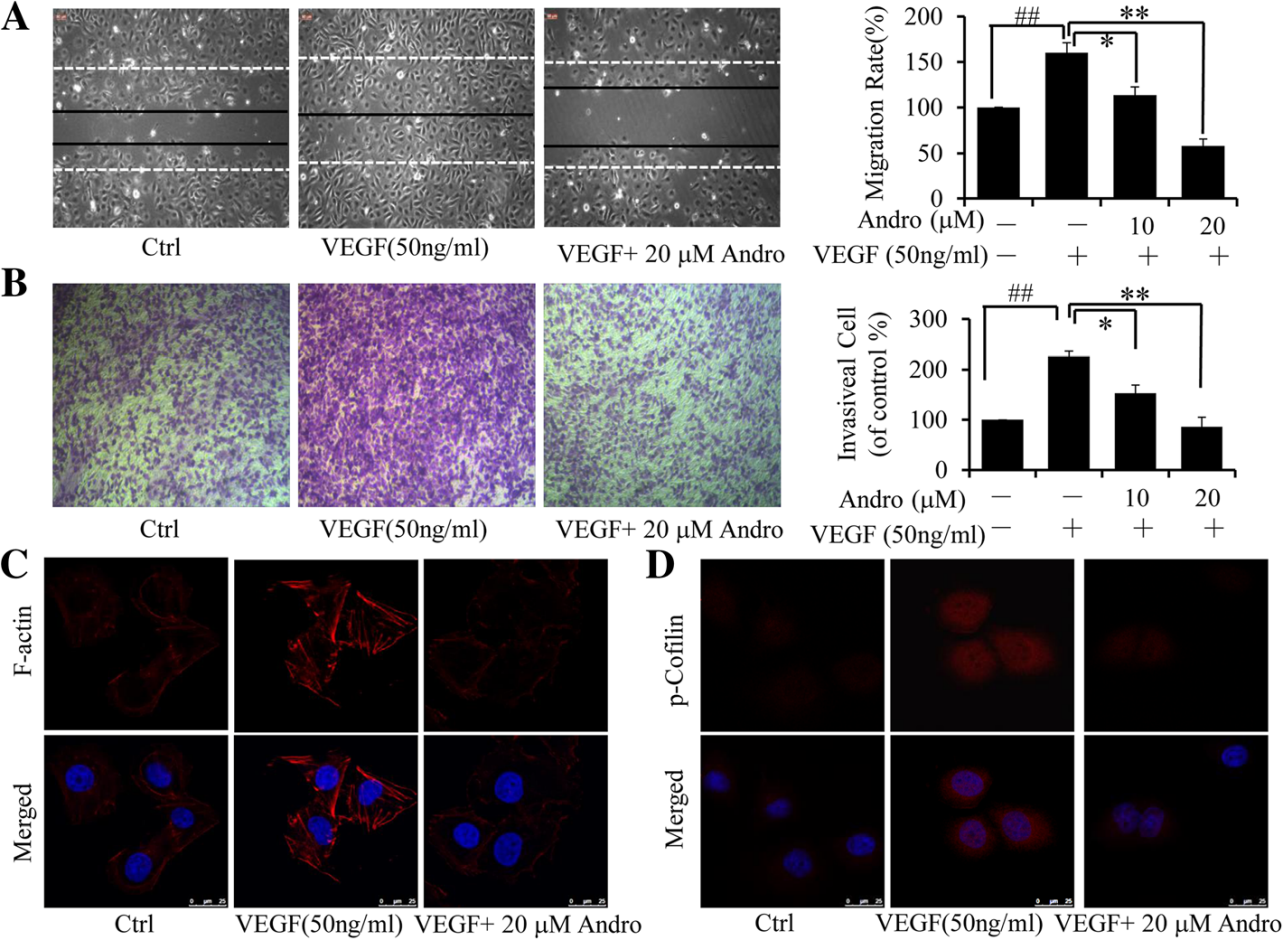
Effect of Andro on VEGF-induced endothelial cell migration and invasion. Endothelial cell migration and invasion were analyzed by a wound-healing assay (**a**) and Transwell assay (**b**). HUVECs were plated, scratched and then incubated with Andro for 24 h, along with or without 50 ng/ml VEGF. Cell migration was measured by manual counting. Original magnifcation, 100× (**a**). **b** HUVECs were plated in Transwell pre-coated with matrigel. Cell migrated to the bottom of the membrane were counted by using an inverted microscope. Original magnifcation, 40×. **c** HUVECs were exposed to Andro for 0.5 h, and then stimulated with or without VEGF for 15 min. F-actin of cells was visualized by dyLightTM 554 palloidin staining and imaged by Leica confocal microscopy. **d** Andro inhibited VEGF-induced coflin phosphorylation in HUVECs using immunofluorescences staining with specifc antibody for phosphorylated coflin. Three independent experiments were performed. (##*p* < 0.01, VEGF-treated group vs. Solvent; **p < 0.05, **p < 0.01, VEGF-treated group vs. VEGF and Andro-treated group)

The effect of Andro on the migration and invasion of HUVEC were also determined by wound healing assays and Transwell assays, respectively. The results indicated that Andro dose-dependently suppressed the migrating and invasive properties of VEGF-induced HUVECs (Fig. 7a and b). Furthermore, we also investigated the effects of Andro on cell cytoskeleton and stress fibre formation, which are key cellular events involved in cell migration through immunofluorescence (IF) assay. As shown in Fig. 7c, VEGF caused a robust induction of stress fiber formation, while this effect could be blocked by treatment of Andro. Besides, it is known that the activation of cofilin is an essential component of actin polymerization and depolymerization, and could play an important role in cell cytoskeleton reorganization. Then we assessed the effects of Andro on VEGF-induced phosphorylated cofilin by using IF analysis. Results indicated that Andro could inhibit VEGF-induced cofilin phosphorylation and activation, suggesting that Andro could inhibit VEGF-triggered HUVECs motility by affecting cofilin activity and the formation of cell stress fiber.

### Andro inhibited tumor growth and tumor angiogenesis in vivo

**Fig. 8:**
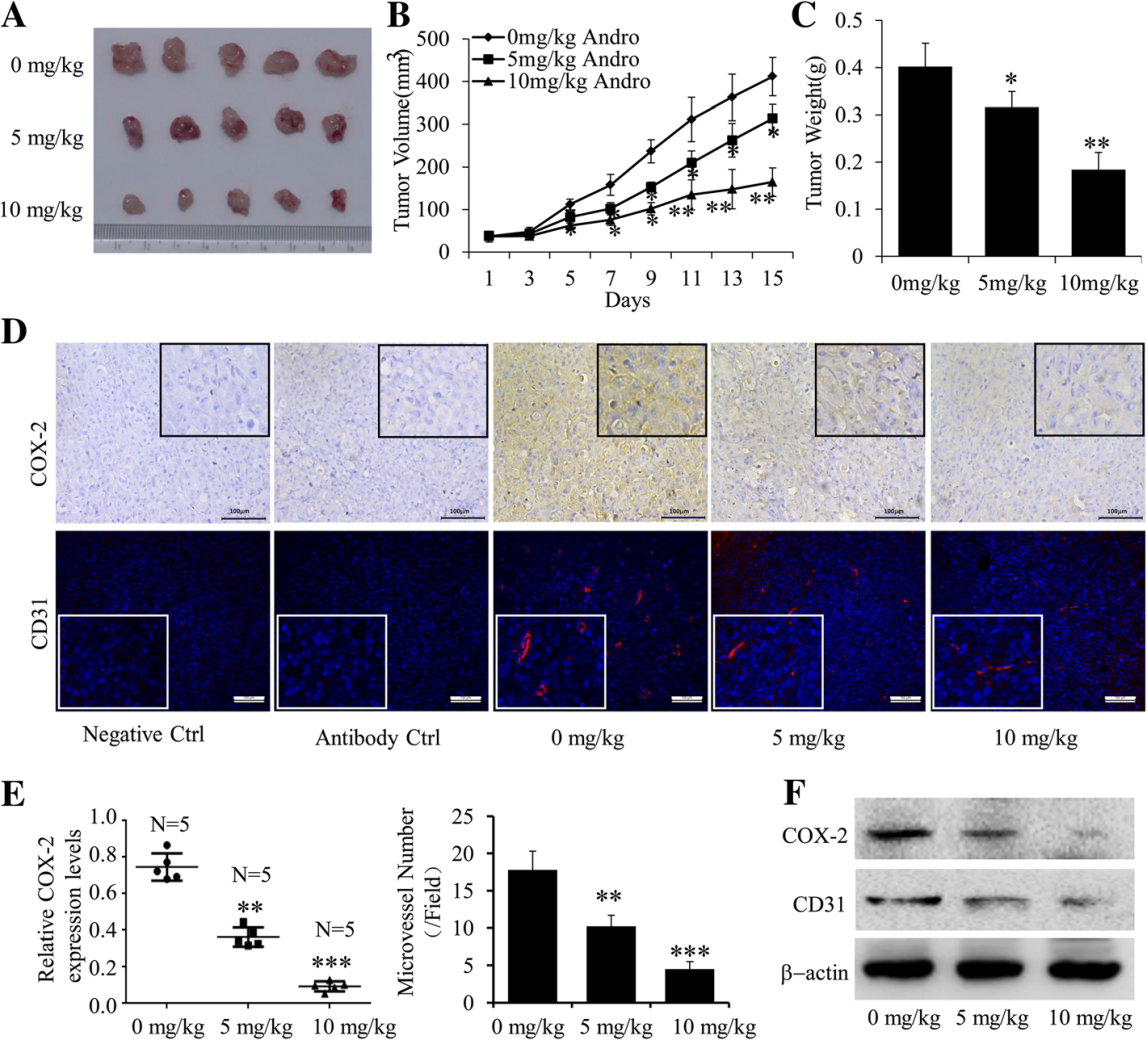
Effect of Andro on tumor growth and tumor angiogenesis in a breast cancer mouse model. An orthotopic mouse model of human breast cancer MDA-MB-231 cells was used to evaluate the anti-tumor effect of Andro. The tumor pictures (**a**), tumor volumes (**b**) and total weights (**c**) were measured. **d** The expressions of COX-2 and CD31 in tumor samples were analyzed by immunohistochemistry and cofocol immunofluorescence, respectively. **e** The quantitative analysis of relative COX-2 expression and microvessel number were also performed**. f** The expression of COX-2 and CD31 proteins in tumor tissues was analyzed by Western blot. Data were represented as the mean ± S.D. (*P < 0.05, **P < 0.01, Andro treatment vs vehicle control groups, *N* = 5 mice/group. Magnification, 200×)

Based on the results of in vitro studies, human breast cancer *xenograft* nude mice model were established to further explore the growth inhibitory effect of Andro in vivo. As expected, as shown in Fig. 8a, b and c, Andro dramatically reduced the tumor volume and the tumor weights, compared with the control group. Furthermore, to determine the precise mechanisms, we further detected the expression of COX-2 in transplanted tumors by using immunohistochemistry (IHC) analysis. As shown in Fig. 8d, when probed with COX-2 antibody, there was a predominantly positive staining in the *xenografts* from control group, and staining intensities become weaker in the treatment group with increasing dose of Andro. In addition, the Western blot analysis of the *xenograft* tumors also indicated that Andro dramatically suppressed COX-2 expression (Fig. 8e). In order to evaluate the effects of Andro on tumor angiogenesis; we have also analyzed the microvessel density through immunostaining of CD31 in *xenograft* tumors by using western blot and confocal immunofluorescence analysis. As shown in Fig. 8d, e and f, the microvessel number and expression levels of CD31 were significantly decreased after the treatment of Andro, compared with the control group. Therefore, these results may indicate that Andro could suppress the breast cancer growth and tumor angiogenesis in vivo.

## Discussion

Andrographolide (Andro) has been reported to be of therapeutic effect for the treatment of various diseases [38, 39]. It has been reported that Andro possessed various biological activities, such as hepatoprotective [40], anti-HIV virus [41], antimalarial [42] antibacterial [43], anti-inflammatory properties [18, 19]. The anti-cancer effect of Andro attracts more and more attention in recent years [44–46], and previous studies have demonstrated that Andro exerted inhibitory effect on human breast tumor growth in vivo and in vitro [20, 21]. In addition, inhibiting HIF-1 and its upstream PI3K/AKT signaling, and inducing G1 arrest and cell apoptosis could all contribute to Andro-induced inhibition of the growth of breast tumor [21, 47]. However, whether there are other signals that involved in the inhibitory effect of Andro still remain unclear.

In China, Andro are prepared as tablets in clinic for anti-bacterial, anti-inflammatory, upper respiratory tract infection and bacterial dysentery. For adult, 2–3 tablets (0.1–0.15 g) were taken at one time, 3–4 times/day, which is about 6.43 mg/kg. The 12.33 fold of human dose equals to the dose of mice, which is a simple method for converting the dosage from human to mice [48]. Converting to dose in mice is about 79.3 mg/kg in oral. And previous study showed that the oral bioavailability of Andro in mice was 9.27 ± 1.69% [49]. Thus, with this conversion and calculation from the oral human dosage, the *i.p.* dosage should be 7.37 mg/kg. For the security, we set 5 and 10 mg/kg for the animal studies according to clinical use, which is for anti-bacterial, anti-inflammatory, upper respiratory tract infection and bacterial dysentery, and there were no obvious toxic effects were detected in mice treated with Andro.

In current study, we have evaluated the response of human breast cancer cells to Andro treatment. Our results indicated that Andro significantly suppressed breast cancer cells growth and induced cell apoptosis through down-regulating COX-2 expression, as well as the activation of COX-2 promoter. Besides, we also found that Andro effectively inhibited COX-2-mediated angiogenesis in human HUVEC cells through suppressing the survival, migration, tube formation and endothelial cell sprouting, which are key steps of angiogenesis. Further explorations showed that Andro-mediated suppression of COX-2 was mainly mediated via inhibiting p300 HAT activity, thereby abrogating the acetylation of trans-activators as well as their binding to COX-2 promoter region in human breast cancer cells. To our knowledge, we are the first to find that Andro could target p300 signaling pathway to regulate COX-2 expression and COX-2-related angiogenesis in human breast cancer cells.

Recently, the carcinogenic properties of chronic inflammation have been recognized as an emerging hallmark of various cancers. COX-2, an inducible form of the COX enzymes, had been reported to be increased in a variety of human cancers [10, 50, 51], including human breast cancer. In current study, we have revealed the precise molecular mechanisms by which Andro inhibited COX-2 expression and COX-2-mediated angiogenesis in human breast cancer. Unlike previous studies, our results indicated that Andro-mediated COX-2 suppression was specifically via targeting p300 signaling pathway. Our study demonstrated that Andro specifically inhibited p300 HAT activity, and abrogates the acetylation of NF-κB p50 and their binding to COX-2 promoter region in human breast cancer.

Furthermore, it is known that COX-2 expression could be mediated by its binding with multiple trans-activators to enhancer elements of its promoter, such as CREB-2, AP-1 (C-Fos) and NF-κB. Besides, p300, the transcriptional coactivator, could increase the transcriptional activity of the NF-κB through directly promoting the acetylation of NF-κB p50 to augment COX-2 transcription [33]. In current study, we used a biotin-labeled DNA probe corresponding to COX-2 promoter region, to investigate the effects of Andro on the transcription activation of COX-2. Our results demonstrated that Andro could siginificantly suppress the binding of muliple trans-activators to the COX-2 promoter, including the CREB-2, C-Fos and NF-κB, thereby inhibiting the transcriptional activation of COX-2. Besides, the promoter activity was also inhibited by Andro in MCF-7 cells, which showed low-level expression of COX-2. That’s because although MCF-7 cells had low-level expression of COX-2, COX-2-related transcription factors, such as NF-κB p65/p50, C-Fos and CREB2, could still express in MCF-7 cells (Fig. 4e). When we transfected an exogenous luciferase expression vector containing COX-2 promoter, these factors could also bind to COX-2 promoter and promote its activity. It is similar for the streptavidin-agarose pulldown assay. In addition, the results also suggested that Andro could inhibit p300 recruitment to the COX-2 promoter.

In the present study, our results indicated that Andro-induced toxicity to breast cancer cells is associated with COX-2. However, we also found Andro-induced apoptosis in MCF-7. The responsible reason was that Andro suppressed COX-2 expression through inhibiting p300 HAT activity and attenuating the acetylation and binding of trans-activators. Although MCF-7 cells had low-level expression of COX-2, Andro could still suppress these targets, such as *NF-κB*. Studies have shown that NF-κB signaling was related to apoptosis. That is to say, high activity of NF-κB signaling could cause anti-apoptosis in cancer cells [52–54]. Therefore, suppression of NF-κB signaling was probably responsible for Andro-induced apoptosis in MCF-7, which could also inhibit COX-2 expression.

It is known that COX-2 is an important regulator of angiogenic milieu, and it stimulates tumor angiogenesis by elevating the expression of vascular endothelial growth factor (VEGF) [34, 55]. Inhibiting COX-2 could lead to angiogenesis suppression, cell growth suppression and apoptosis induction [56]. Results from previous studies have revealed that COX-2 inhibitors could effectively suppress angiogenesis and tumor growth, prevent metastasis, and increase overall survival [56]. In our study, for the first time, we demonstrated the effect of Andro on COX-2 expression and angiogenesis. These results suggested that Andro effectively inhibited the angiogenesis of human HUVECs, including the survival, migration and invasion, tube formation and endothelial cell sprouting.

## Conclusion

In the present study, our results fully demonstrated that Andro could suppress the proliferation and angiogenesis of breast cancer cells through suppressing the p300/COX-2 and VEGF signaling pathway. Our findings have highlighted the potential inhibitory effects of Andro on human breast cancer and have also revealed its precise molecular mechanisms of anti-cancer. Above all, Andro could be used as an effective and safe agent for breast cancer therapy in near future.

## Abbreviations

Andro: Andrographolide
CB: Celecoxib
CFSE: Carboxyfluorescein succinimidyl ester
ChIP: Chromatin immunoprecipitation
COX-2: Cycloxygenase-2
cyt c: Cytochrome c
NF-κB: Nuclear Factor κB
p300 HAT: Histone acetyltransferase p300
VEGF: Vascular endothelial growth Factor
