# Correction: Andrographolide inhibits breast cancer through suppressing COX-2 expression and angiogenesis via inactivation of p300 signaling and VEGF pathway

**DOI:** 10.1186/s13046-023-02818-7

**Published:** 2023-09-16

**Authors:** Yulin Peng, Yan Wang, Ning Tang, Dongdong Sun, Yulong Lan, Zhenlong Yu, Xinyu Zhao, Lei Feng, Baojing Zhang, Lingling Jin, Fabiao Yu, Xiaochi Ma, Chuanzhu Lv

**Affiliations:** 1 Institute of Integrative Medicine, College of Pharmacy, College of Basic Medical Science, Dalian Medical University, Dalian, 116044 China; 2 Emergency Department, The Second Affiliated Hospital of Hainan Medical University, Haikou, 571199 China; 3 Department of Integrative Medicine, Liaoning University of Traditional Chinese Medicine Xinglin College, Shenyang, 110167 China

**Correction:** ***J Exp Clin Cancer Res*** **37, 248 (2018)**

10.1186/s13046-018-0926-9

Following publication of the original article [1], errors were found in Figs. 6 and 8. The band of β-actin in Fig. 6B (Basal) and the band of CD31 in Fig. 8F were mistakenly uploaded.

The corrected figures are provided below:

**Fig. 6:**
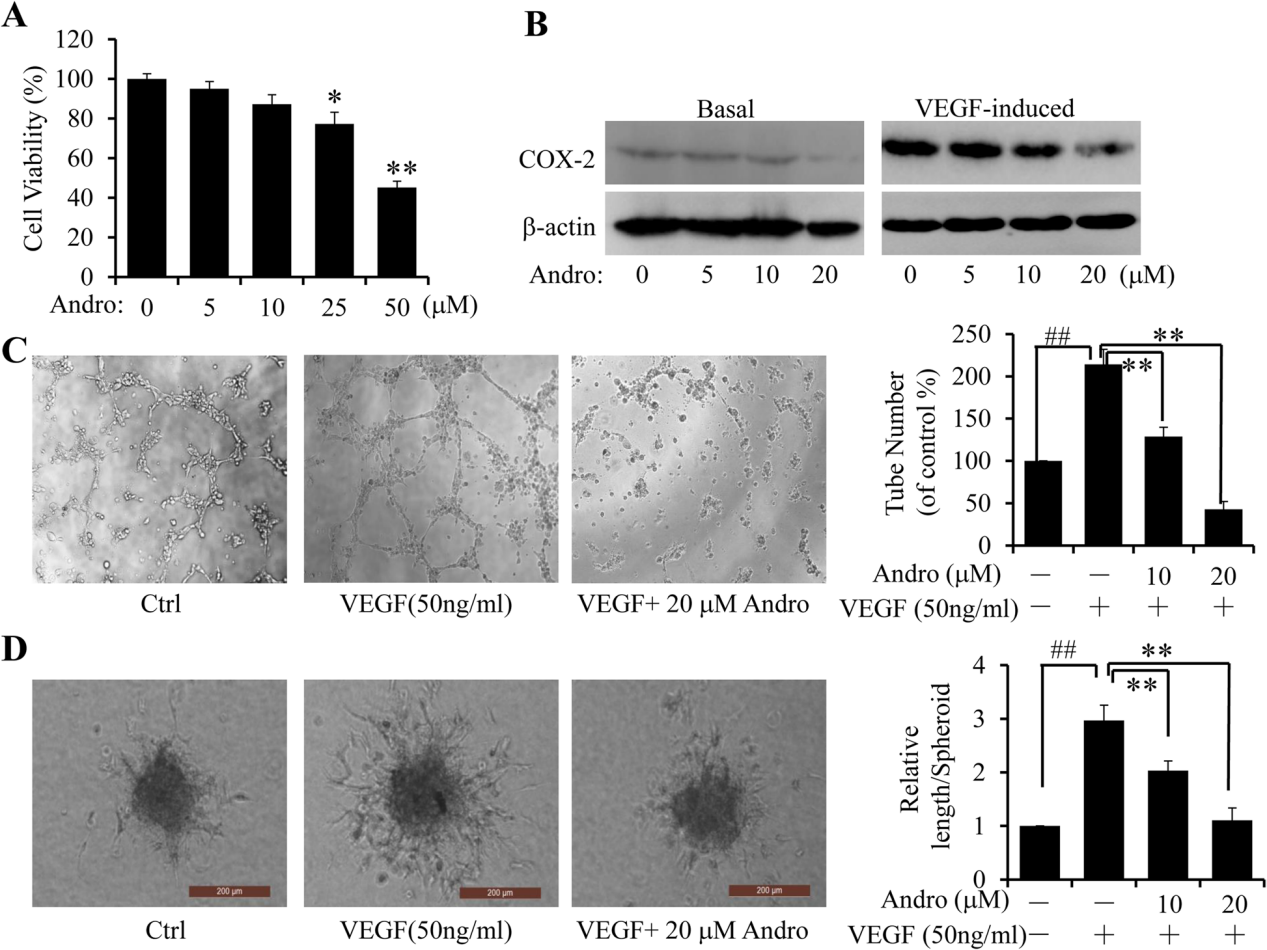
Effect of Andro on VEGF-induced angiogenesis. **a** HUVECs were exposed to Andro at the indicated doses, and viability was measured by CCK-8 assay. Data were represented as percentage of vehicle-treated control. **b** The expression level of COX-2 protein was analyzed by Western blot HUVECs treated with the indicated doses of Andro for 48 h, with or without VEGF induction. **c-d** Effects of Andro on tube formation on Matrigel c at 6 h (Original magnifcation, 50 ×), and sprouting from modifed human endothelial cell spheroids **d** at 24 h (Original magnifcation, 200 ×). Experiments were performed with or without VEGF and indicated Andro doses. (##*p* < 0.01, VEGF-treated group vs. Solvent; *P < 0.05, **P < 0.01, Andro treatment vs vehicle control groups)

**Fig. 8:**
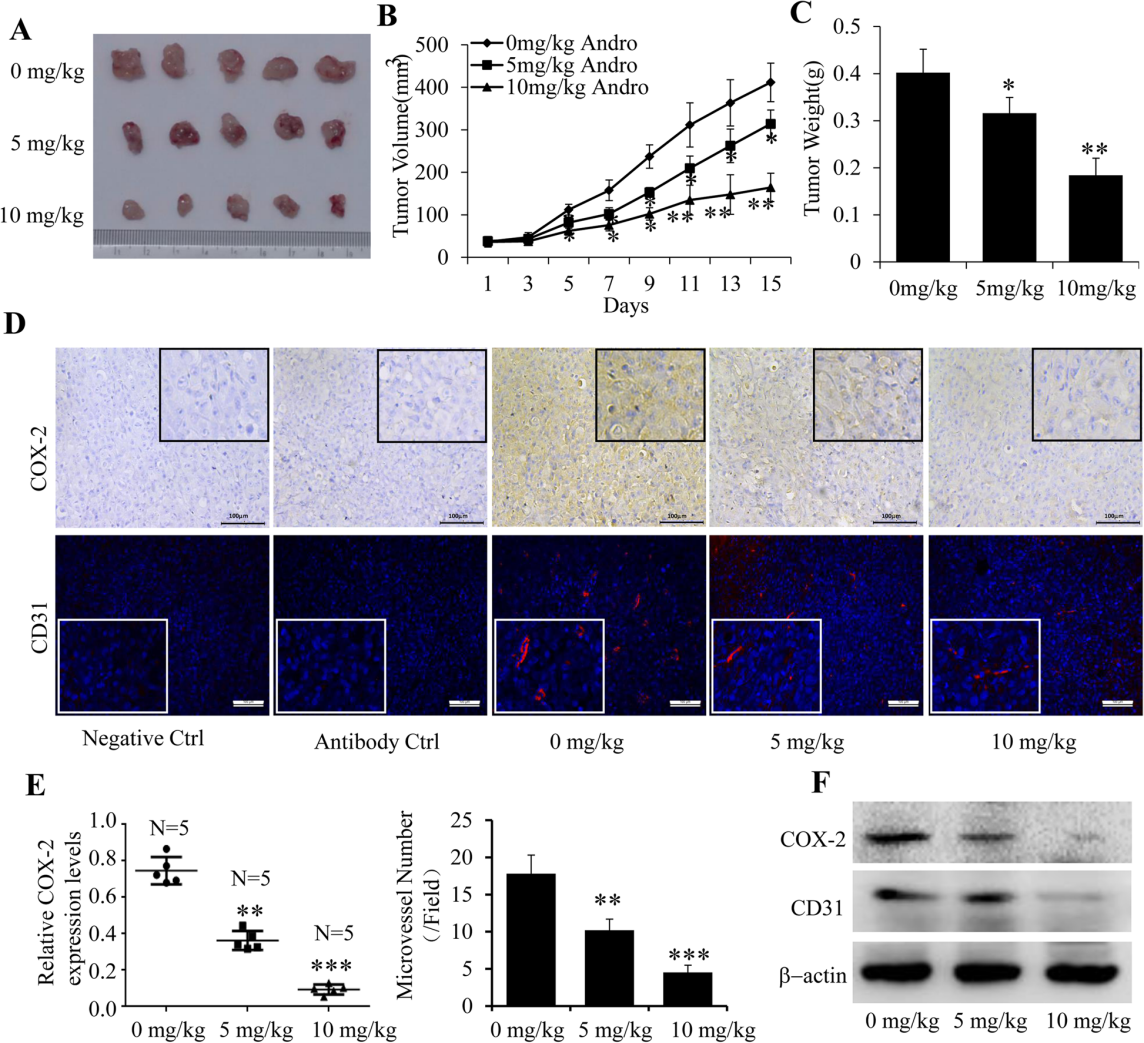
Effect of Andro on tumor growth and tumor angiogenesis in a breast cancer mouse model. An orthotopic mouse model of human breast cancer MDA-MB-231 cells was used to evaluate the anti-tumor effect of Andro. The tumor pictures (**a**), tumor volumes (**b**) and total weights (**c**) were measured. d The expressions of COX-2 and CD31 in tumor samples were analyzed by immunohistochemistry and cofocol immunofluorescence, respectively. **e** The quantitative analysis of relative COX-2 expression and microvessel number were also performed. **f** The expression of COX-2 and CD31 proteins in tumor tissues was analyzed by Western blot. Data were represented as the mean ± S.D. (*P < 0.05, **P < 0.01, Andro treatment vs vehicle control groups, *N* = 5 mice/group. Magnification, 200 ×)

The corrections do not affect the overall result, discussion, or conclusion of the article.

## References

[1] Peng Y, Wang Y, Tang N (2018). Andrographolide inhibits breast cancer through suppressing COX-2 expression and angiogenesis via inactivation of p300 signaling and VEGF pathway. J Exp Clin Cancer Res.

